# Effects of Pharmacological Treatment on Telomere Length and the Expression of Telomerase/Shelterin-Related Genes in Rat Models of Autism

**DOI:** 10.1007/s12031-025-02353-4

**Published:** 2025-04-24

**Authors:** Elena V. Valeeva, Dmitry O. Nikitin, Lubov S. Nikiforova, Irina I. Semina, Ildus I. Ahmetov

**Affiliations:** 1 Central Research Laboratory, Kazan State Medical University, Kazan, 420012 Russia; 2 Laboratory of Genetics of Aging and Longevity, Kazan State Medical University, Kazan, 420012 Russia; 3 Pharmacology Department, Kazan State Medical University, Kazan, 420012 Russia; 4 Research Institute for Sport and Exercise Sciences, Liverpool John Moores University, Liverpool, UK

**Keywords:** Autism, Telomere, Expression genes, Amitriptyline, Risperidone, Nooclerin

## Abstract

Telomeres are increasingly recognized for their potential role in the etiology of autism spectrum disorder (ASD) due to their involvement in cellular aging and telomerase-shelterin function. Although shorter telomeres have been observed in individuals with ASD, studies linking telomere dynamics in blood cells and brain regions remain limited. Using the valproic acid (VPA, 500 mg/kg) rodent model, this study aimed to assess the impact of three drugs commonly used in ASD treatment (amitriptyline, risperidone, and nooclerin) on telomere length and the expression of telomerase/shelterin-related genes (*Dkc1*, *Gar1*, *Pot1a*, *Pot1b*, *Tep1*, *Terc*, *Terf2ip*, *Tert*, *Tinf2*, *Tnks*, *Tpp1*, *Trf1*, and *Trf2*) in blood cells, the prefrontal cortex, and hippocampus of VPA-exposed Wistar rats. Telomere length and gene expression were measured using quantitative PCR. Risperidone treatment in VPA males resulted in telomere elongation and increased expression of *Tnks* in blood cell and *Trf1*, *Trf2* genes in prefrontal cortex. Nooclerin treatment also showed beneficial effects on telomere length of blood cell in males, alongside increased *Trf1* expression. Long telomeres in male blood cells were associated with reduced anxiety, while a positive correlation was found between *Tpp1* expression and stereotypical behavior in both male and female VPA rats. These findings suggest that nooclerin and risperidone influence telomere length and gene expression related to the telomere-telomerase complex in a sex-dependent manner, offering insights into the neurobiological mechanisms underlying ASD.

**Supplementary Information:**

The online version contains supplementary material available at 10.1007/s12031-025-02353-4.

## Introduction

Telomeres, composed of repeating nucleotide sequences (TTAGGG)_n_ and associated with shelterin proteins, are essential for maintaining genome integrity by protecting chromosome ends from degradation (Blackburn 2005). While somatic cells experience telomeric DNA loss with each replication cycle, this erosion is prevented in germ cells, stem cells, and cancer cells due to telomerase activity (Lansdorp 2022; Shay & Wright 2011). Critically short telomeres can trigger apoptosis and cellular senescence (Mathon & Lloyd 2001). Various genetic, epigenetic, and environmental factors influence telomere erosion rates (Mukherjee et al. 2018; Mirabello et al. 2010; Buttet et al. 2022), and multiple studies have linked telomere shortening to various pathologies (Wang et al. 2018; Ridout et al., 2016; Polho et al. 2015; Zeng et al. 2020; Xu et al. 2019). However, research on telomere length in individuals with autism spectrum disorder (ASD) is limited, particularly regarding its relationship with specific clinical symptoms.

ASD is a neurodevelopmental pathology that is characterized by specific early social and communicative deficits, as well as an interest restriction (Sharma et al. 2018; Semina et al. 2019; Hirota & King 2023). ASD encompasses a diverse range of disorders, including infantile autism, Asperger’s syndrome, atypical autism, and others. This heterogeneity presents a significant challenge for accurate diagnosis and effective treatment (Maenner 2021; Sharma et al. 2018). The current estimate is that ASD is diagnosed in one in 36 children (Maenner 2021). The prevalence of ASD is higher among males (1:4) (Maenner 2021). However, ASD is much more severe in females, with severe socio-communicative impairment, irritability, lethargy, poor adaptive skills, and low cognitive abilities (Frazier et al. 2014; Rynkiewicz et al. 2016; Solomon et al. 2012). The role of telomeres and telomerase activity in the etiology of ASD remains unclear. Despite recent research, there is currently limited evidence to suggest a link between telomere length and telomerase subunit activity and this disorder (Panahi et al. 2023; Li et al. 2014; Lewis et al. 2020; Lin et al. 2022). One of the proposed mechanisms for telomere shortening in patients with ASD is linked to oxidative stress (Zhang et al. 2023).There is a lack of animal studies investigating telomere length in autism models. One common model involves administering valproic acid (VPA) to fetal rats to induce autism-like symptoms. VPA affects glutamatergic differentiation in the embryonic rat brain by altering Pax6 transcription, leading to increased TERT expression through histone deacetylase inhibition (Kim et al. 2014; Kim et al. 2017). However, more research is needed to understand the molecular changes in the telomere-telomerase system in offspring exposed to VPA prenatally. To date, there are no effective treatments ASD and the pharmacological interventions currently available often come with significant side effects (Kennedy & Adolphs 2012). ASD patients typically experience challenges in social interaction, spatial reasoning, memory, and emotional regulation—functions that are primarily supported by the hippocampus and prefrontal cortex. Certain medications used in treating ASD may target these cognitive and emotional processes, potentially enhancing the functioning of these critical brain structures and leading to improvements in behavior. Furthermore, these pharmacological agents may also influence telomere length, a factor that plays a crucial role in cellular aging and overall health. The study of telomere length can facilitate the identification of alterations in cellular processes occurring in response to pharmacological agents, thereby enhancing the assessment of their safety and predicting the efficacy of treatment.

The objective of the study was to investigate the impact of pharmacological agents employed in the treatment of ASD, namely the antidepressant amitriptyline, the antipsychotic risperidone, and the nootropic agent nooclerin, on telomere length and the expression of telomerase/shelterin-related genes.

## Methods

### Animals

Prior to the commencement of the experiment, all rodents were cared for in accordance with the guidelines set forth for the treatment of laboratory animals. The rodents were housed at a temperature of 21 ± 2 ℃ and a light cycle consistent with the European Convention for the Protection of Vertebrate Animals (Strasbourg, 1986). Furthermore, the animals were provided with a complete diet that met the nutritional standards set forth in the convention. The study was approved by the local ethics committee of the Kazan State Medical University of the Ministry of Health of the Russian Federation (protocol no. 10, 19–10–2017).

The studies were conducted on 49 female (170–220 g) and 49 male (200–300 g) Wistar rats aged 90 ± 5 days. The rats were obtained from the Stolbovaya laboratory animal nursery, which is located in the Moscow region.

**Fig. 1:**
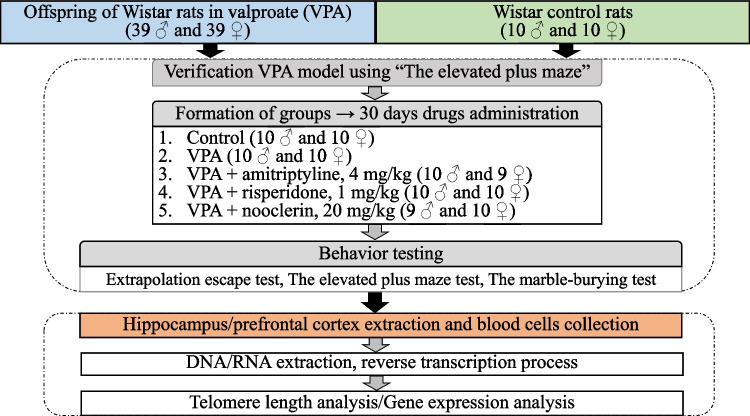
Experimental design

The modelling of fetal valproate syndrome in animals was conducted through the subcutaneous administration of valproic acid (Convulex; G.L. PHARMA, Austria) at a dose of 500 mg/kg to pregnant females on the 13 th day of gestation (Schneider & Przewłocki 2005). The experiments were conducted on the offspring of 78 rats (39 males and 39 females), with 10 males and 10 females constituting the VPA group. The remaining 58 rats in the valproate model of autism were randomly assigned to one of the following groups of rodents, which were administered drugs used in the treatment of ASD for a period of 30 days. The antidepressant amitriptyline was administered at a dose of 4 mg/kg (Moscow Endocrine Plant, Moscow). The antipsychotic risperidone was administered at a dose of 1 mg/kg (Janssen Pharmaceuticals, Belgium), while the drug with nootropic effect nooclerin was administered at a dose of 20 mg/kg (Pik-PHARMA, Moscow) (Fig. 1).

A control group consisting of 10 females and 10 males of Wistar rats of the same age was also included in the study. These individuals were administered a saline solution of the same volume during the same period as the experimental group.

### Behavior

The development of fetal valproate syndrome was verified through the utilization of the behavioral testing method designated as the elevated plus maze (EPM). The EPM test is employed as a means of evaluating the potential degree of anxiety exhibited by an animal (Walf & Frye 2007). The design comprises a platform elevated 70 cm from the floor (14 × 14 cm) with four perpendicularly oriented corridors or sleeves (50 × 14 cm). Two of the close arms (analogous to a hole) and two are open arms (potentially dangerous zones for the animal). The test is conducted over a period of 3 min for each rodent. At the outset of the experiment, the rat is positioned in the center of the labyrinth with its muzzle oriented towards the open corridor. Subsequently, the time spent by the animal in the open arms of the labyrinth is recorded. A decrease in this time is considered an indicator of anxiety development.

The registration of the EPM test was conducted utilizing the EthoVisionXT (11.5 version) software (Noldus, Netherlands) with the incorporation of automated track recording.

Following a 30-day period of administration of the study drugs, behavioral testing was conducted using the following tests (Fig. 1).

The Extrapolatory Escape test enables the assessment of behavior in the context of acute stress conditions (Bondarenko 2017). The animal under study was placed inside a cylinder, the limited water space of which is considered to be a high-stress environment. The animal was unable to escape this environment by any other means than by diving under the lower edge of the cylinder. During the 2-min test, the duration of the jumping period (in seconds) was taken into account. A longer jumping period indicated a higher anxiety level (Bondarenko 2017).

The Marble Burying test is predicated on the assumption that aberrant burying may be a manifestation of neophobic and stereotypical behavior, characterized by an aversion to novel or unfamiliar objects (Angoa-Pérez et al. 2013). A standard cage (40 × 20 × 20 cm) was filled with sawdust to a depth of 3 cm, after which 20 glass balls (*d* = 1.6 cm) were placed on the surface at equal distances. The test animal was then placed in the cage and left alone for 30 min. Subsequently, the rat was removed, and the number of intact balls was counted. It was observed that an increased number of intact balls buried compared to the control animals corresponded with a more pronounced stereotypy (Dixit et al. 2020). During the experiment, each animal was tested on three occasions. For the purposes of this study, balls that were hidden by sawdust by more than two-thirds were considered to be buried.

### Collection of Tissues

On the day following the final behavioral testing, whole blood was collected from the tail vein of rats in a volume of 1 mL into a test tube containing 1.2 mg/mL EDTA-K2 solution. On the following day, decapitation and the extraction of individual brain structures (hippocampus, prefrontal cortex) into test tubes was conducted, after which the test tubes were placed in a freezer at − 80 °C until analysis.

### The Estimation of Relative Telomere Length

Genomic DNA was isolated from blood cells, hippocampus and prefrontal cortex according to the protocol of the used commercial kit (diaGene, Russia). The quantity and quality of DNA from the extracted samples was measured using a NanoDrop Lite spectrophotometer (ThermoFisher, USA). The concentration of genomic DNA in the blood was 34.4 ± 4.6 ng/μL, in the hippocampus 167.7 ± 6.9 ng/μL and in the prefrontal cortex 65.4 ± 4.4 ng/μL.

For real-time quantitative PCR analysis, 20 ng DNA, 2 × Biomaster HS-qPCR SYBR Blue mix (Biolabmix, Russia), 200 nM of each primer (*Gapdh* F: 5′-cgaccccttcattgacctcaactac- 3′, *Gapdh* R: 5′-cactccaccacatactcagcaccg- 3′; *Tel1* F: 5′-ggtttttgagggtgagggtgagggtgagggtgagggtgagggt- 3′, *Tel1* R: 5′-tcccgactatccctatccctatccctatccctatcccta- 3′) and nuclease-free water were used. PCR amplification conditions were 95 °C for 5 min, 30 cycles of 95 °C for 10 s, 63 °C for 30 s with detection of SYBR fluorescence signal and subsequent melting curve analysis. The mixture with each DNA sample was performed in triplicate. The calculation of T/S, reflecting the ratio of the telomeric sequence to the single-copy gene, expressed in the formula 2^−ΔCt^, and the relative telomere length (2^−ΔΔCt^) was carried out according to the original method of Cawthon (2002).

### RNA Isolation and Reverse Transcription

Total RNA was isolated from each rat structure using the ExtractRNA (BC032, Evrogen, Russia) and Lysing Matrix D ceramic beads (*d* = 1.4 mm) (MP Biomedicals, USA) according to the manufacturer’s instructions. Blood cells were also isolated without the homogenization step using ExtractRNA (Moscow, Russia). The amount of RNA from the extracted samples was measured using NanoDrop Lite spectrophotometer (ThermoFisher, USA). Reverse transcription was conducted using an RNA template of 2 μg, M–MuLV–RH reverse transcriptase, 5 × RT-buffer-mix, 2 μL of random primers and sterile water, in accordance with the protocol provided by the manufacturer (Biolabmix, Russia).

### Estimation of Gene Expression

The resulting cDNA samples were then subjected to real-time qPCR on a CFX96 (BioRad, USA) using synthesized primers for *Dkc1*, *Gar1*, *Pot1a*, *Pot1b*, *Tep1*, *Terc*, *Terf2ip*, *Tert*, *Tinf2*, *Tnks*, *Tpp1*, *Trf1*, *Trf2* gene (sequence primers in the Supplementary Table 1) and the *18 sRna* as reference gene and with 2 × Biomaster HS-qPCR SYBR Blue mix (Biolabmix, Russia). Each analysis was performed in triplet. Gene expression levels were calculated using the formula 2^−ΔΔCt^ (Livak & Schmittgen 2001) relative to the control group of healthy saline-treated rats.

### Statistical Analysis

Statistical analyses were performed using GraphPad Prism 8.0.1 software (GraphPad Software, Inc., San Diego, CA, USA) with one-way ANOVA analysis of variance. Telomere behavior and relative length results are presented as mean ± standard error of the mean (SEM). The critical threshold for significance was *p* < 0.05.

## Results

**Fig. 2:**
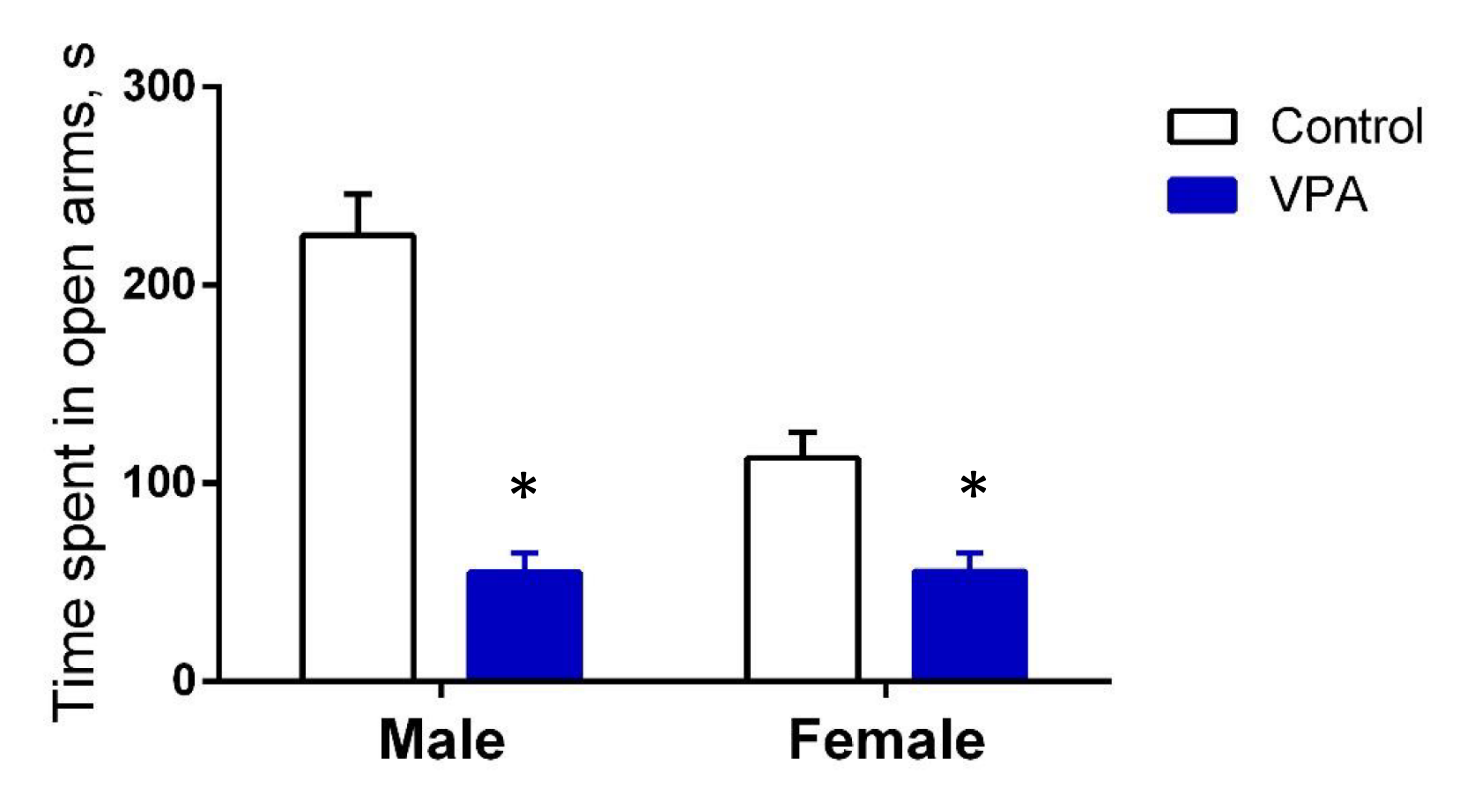
The average time (mean ± SEM, s) spent rats in open arms of the elevated plus maze. *p < 0.001

In order to validate the constructed valproate model of autism, the EPM was employed as a conventional test, which demonstrated that the rats utilized in the autism model exhibited elevated anxiety prior to the administration of drugs. In both male and female VPA rats, there was a notable reduction in the time spent in the open arms of the EPM when compared to the control group (*p* < 0.001). This observation is indicative of an elevated state of anxiety in the VPA rats (Fig. 2).

### The Sexual Dimorphism of T/S in VPA Rats

A statistically significant difference of T/S was observed in the hippocampus of healthy male rats and VPA rats (reflecting the ratio of telomeric sequence to single-copy gene) being higher in males compared to females (Fig. 2, *p* < 0.0001). In contrast, an increase in the T/S ratio was observed in females compared to males in the study groups in the prefrontal cortex (Supplementary Table 2, *p* < 0.05). The T/S length ratio in the blood cells of females in the control group was higher than that of males (*p* = 0.003). Nevertheless, no statistically significant difference between the sexes was identified in the VPA group (*p* > 0.05). It can be posited that future comparative assessments of telomere length in rats should be conducted separately for males and females.

### Comparative Assessment of Relative Telomere Length

**Fig. 3:**
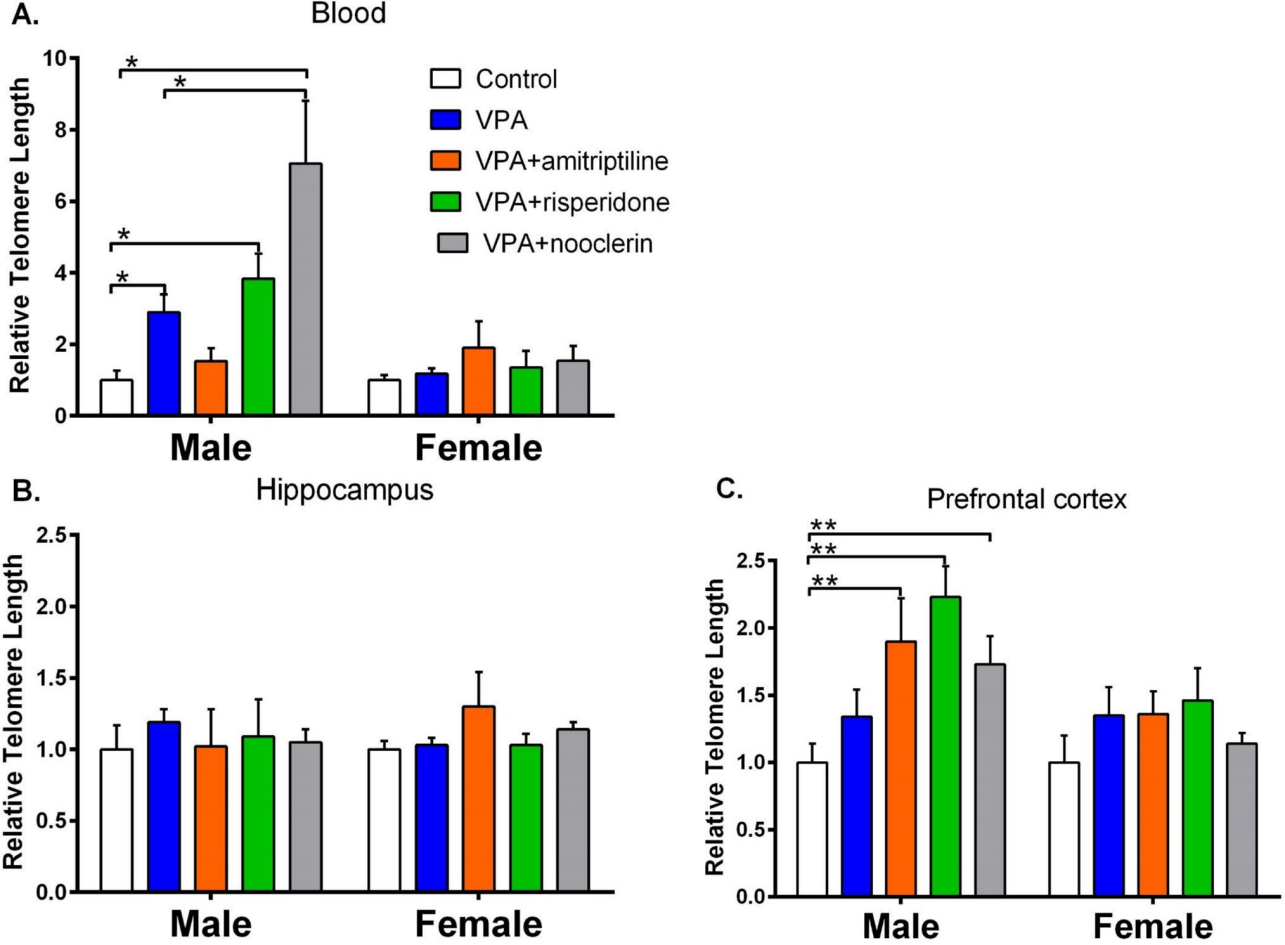
Effect of amitriptyline, risperidone and nooclerin on telomere length (mean ± SEM) in blood cells (**a**), hippocampus (**b**), and prefrontal cortex (**c**) in VPA rats relative to the control group. *p ≤ 0.005, statistically significant differences relative control; **p ≤ 0.0001, statistically significant differences relative VPA

A comparative analysis of the relative telomere length (RTL) of VPA male’s rats revealed that the RTL value in blood cells was higher than in the control group (*p* = 0.002, Fig. 3). No significant difference was observed in the cells of the prefrontal cortex and hippocampus in females and males in the autism model when compared to the control (*p* > 0.05). Moreover, the impact of pharmacological agents on RTL was assessed. The administration of nooclerin in the male rats, when compared to the VPA group without treatment, resulted in a notable elevation in this parameter in the blood, reaching a value approximately twofold higher (2.90 ± 0.50 versus 7.06 ± 1.75, *p* = 0.0031, Fig. 3) and sevenfold higher when compared to the control group (1.00 ± 0.27 versus 7.06 ± 1.75, *p* = 0.0022). In the prefrontal cortex, nooclerin increased RTL by 1.73 times relative to the control group but did not affect VPA (1.00 ± 0.14 vs. 1.73 ± 0.21, *p* = 0.0001). The administration of risperidone resulted in a significant increase in RTL in male blood cells, with a 3.84-fold elevation observed in the VPA (1.00 ± 0.27 vs. 3.84 ± 0.70, *p* = 0.0043) and a 2.23-fold increase in the prefrontal cortex relative to the control (1.00 ± 0.14 vs. 2.23 ± 0.23, *p* < 0.0001). The administration of amitriptyline to VPA male rats resulted in an increased RTL, as observed in prefrontal cortex cells when compared to the control group (1.00 ± 0.14 vs. 1.90 ± 0.32, *p* < 0.0001; Fig. 3). No statistically significant difference was identified between the groups in the RTL indices of VPA female blood cells, hippocampus, and prefrontal cortex (*p* > 0.05).

### Correlation Analysis of Relative Telomere Length Between Blood, Hippocampal, and Prefrontal Cortex Cells

A significant correlation was identified between the RTL of blood cells, the hippocampus and the prefrontal cortex in males treated with nooclerin (*R* = 0.7998, *p* = 0.0031). However, no such correlation was observed in the other groups of rats. No such correlation was observed in the female subjects.

### Evaluation of Gene Expression of Telomerase Activity Subunits

The transcriptional activity signature of shelterin complex genes (*Trf1 - 2*, *Tinf2*, *Tpp1*, *Pot1a*, *Pot1b*, *Terf1ip*) and genes regulating telomerase activity (*Dkc1*, *Gar1*, *Tep1*, *Terc*, *Tert*), as well as *Tnks*, differs between female and male rats in the valproate model of autism, depending on the type of tissues. the expression level of *Tpp1*, *Pot1a*, and *Tnks* in the hippocampus in female VPA rats was found to be almost threefold lower than that observed in the control group (Supplementary Table 3, *p* = 0.0276, *p* = 0.0422, and *p* = 0.025). The *Tep1* expression level was reduced to RQ 0.37 in blood cells (Supplementary Table 3, *p* = 0.0349). Conversely, an increase in the *Pot1a* and *Gar1* expression genes was observed in blood cells of VPA males compared to the control.

**Fig. 4:**
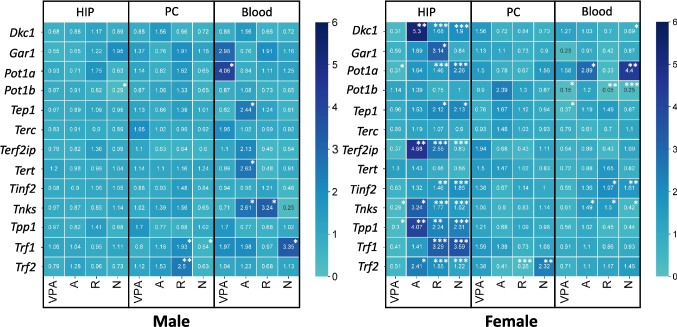
Heatmaps for telomerase subunits gene expression analysis with significantly relative values. *Abbreviations:* A, amitriptyline; R, risperidone; N, nooclerin; HIP, hippocampus; PC, prefrontal cortex. * 0.01 < *p* < 0.05; ** 0.01 < *p* < 0.001; *** 0.001 < *p* < 0.0001

In general, depending on the sex of the VPA rats, significant changes in gene expression were observed in response to the influence of the studied drugs, with notable differences between the sexes. To illustrate, in the hippocampus of VPA males, only nooclerin resulted in a reduction in the expression of the *Pot1b* gene (relative quantification (RQ, 2^−ΔΔCt^) 0.29, *p* = 0.0152). In contrast, in VPA females, the influence of risperidone, amitriptyline, and nooclerin led to an increase expression of telomerase/shelterin-related genes (Fig. 4), except for the *Pot1b*, *Terc*, and *Tert*, which demonstrated no alterations.

The administration of risperidone and amitriptyline to VPA rats resulted in notable alterations in the gene expression of the prefrontal cortex and in blood cells, irrespective of gender. Thus, the administration of risperidone was observed to induce alterations in the *Trf2* expression in the prefrontal cortex of both male and female VPA rats. In the male subjects, the indicator exhibited an increase to RQ 2.50 (*p* = 0.0047), whereas in the female rats, a reduction was noted to RQ 0.26, *p* = 0.0004). In blood cells, risperidone caused an increase in the *Tnks* expression in VPA males (RQ 3.24, *p* = 0.0411) and in VPA females (RQ 1.50, *p* = 0.0276). Furthermore, an increase in the *Tnks* expression was observed when administering amitriptyline in VPA males (RQ 2.61, *p* = 0.0211) and in VPA females (RQ 1.49, *p* = 0.0303) (Supplementary Table 1).

### Correlation of Expression Levels of Telomerase/Shelterin-Associated Genes and Telomere Length With Behavior

A correlation was performed between the expression levels of the studied genes and behavior (Supplementary Table 4). It was anticipated that alterations in behavioral patterns and the direction of gene expression would be observed, with characteristics that were consistent with those typically associated with each sex. Thus, the expression of the genes of the shelterin complex *Pot2a* in the hippocampus and prefrontal cortex of males correlated with the time (s) before diving in the Extrapolation Escape test (*r* = 0.3567, *p* = 0.0096). Furthermore, a negative correlation was observed between the expression of the *Pot2a* gene in blood cells and the data on the time (s) in open arms in the elevated plus maze test (*r* = − 0.4259, *p* = 0.0096, and *r* = − 0.3195, *p* = 0.0286, respectively).

Furthermore, the expression of another shelterin complex gene, *Terf2ip*, and the telomerase subunit of the *Dkc1* gene in the blood of males was found to be significantly correlated with the number of marbles buried (*r* = 0.5128, *p* = 0.0104, and *r* = 0.4121, *p* = 0.0172, respectively). In contrast, in females, only the expression of one shelterin complex gene, *Trf1*, in the prefrontal cortex demonstrated a significant correlation with the number of marbles buried (*r* = − 0.3614, *p* = 0.0357) (Supplementary Table 4). In addition, a notable correlation was observed between the *Tnks* gene and the time in open arms (*r* = 0.4175, *p* = 0.008). It is noteworthy that the expression of the *Tert* gene in the prefrontal cortex of males demonstrated a statistically significant correlation with the time before diving (*r* = − 0.3401, *p* = 0.0193). In females, the correlation between *Tert* expression in the hippocampus and the number of marbles buried was also significant (*r* = − 0.3319, *p* = 0.0448). However, only the *Tpp1* gene, which was expressed in blood cells equally for both females and males, demonstrated a positive and statistically significant correlation with The number of marbles buried (*r* = 0.5259, *r* = 0.0041 for males, *r* = 0.5099, *p* = 0.0129 for females) (Supplementary Table 4).

A correlation analysis of behavioral data with telomere length revealed that in males, blood telomere length was associated with time to dive in the extrapolation escape test (*r* = 0.6414, *p* < 0.0001; Supplementary Table 4). This demonstrated a positive association between long telomeres and reduced anxiety.

## Discussion

The study of the effects of valproic acid during gestation as a model of autism has been demonstrated in numerous rodent studies to be a robust environmental factor. The study of the effects of valproic acid during gestation as a model of autism has been demonstrated to be a robust environmental factor in numerous rodent studies. Some studies have demonstrated that valproic acid (VPA) can induce morphological abnormalities in the cerebellum (Ingram et al. 2000), an imbalance between excitatory and inhibitory neurotransmission (Kim et al. 2013, 2014), as well as histone hyperacetylation and altered MeCP2 expression (Kim et al. 2016; Liu et al. 2021).

Valproic acid has been demonstrated to induce behavioral deviations that are characteristic of patients with ASD (Schneider & Przewłocki 2005). In the initial phase of our investigation, we employed the EPM behavioral test to validate the autism model in rats. Our findings confirmed a correlation between prenatal exposure to valproic acid (VPA) and increased anxiety levels. Notably, previous research has indicated that this heightened anxiety in rats exposed to VPA during prenatal development is primarily observed in males (Semina et al. 2023).

The current state of knowledge regarding the molecular mechanisms underlying sex differences in the risk of developing ASD, as well as the processes associated with these differences, is limited. Furthermore, the presence of sexual dimorphism in telomere length in individuals with autism suggests the involvement of a sex factor (Panahi et al. 2023). The objective of our study was to examine the effect of prenatal administration of valproic acid on telomere length in the offspring of rats of different sexes. It was demonstrated that in the control group of females, telomeres in blood cells exhibited significantly greater length than those observed in males. The findings are in accordance with the data presented in a systematic review, which confirmed a significant association between long telomeres in blood cells in women (Gardner et al. 2014) and can be explained by several factors. The initial rationale pertains to the favorable impact of estrogen on telomerase activity in blood cells (Aviv 2002), which results in telomere elongation. The second rationale is based on the heterogametic sex hypothesis, which posits that men (carriers of XY) will exhibit a greater prevalence of deleterious recessive alleles compared to women (Barrett & Richardson 2011). This, in turn, can influence the length of telomeres in blood cells due to the diminished transcriptional activity of certain genes that are capable of protecting telomeres. Subsequently, we demonstrated that although prenatal administration of VPA solely to blood cells resulted in an increase in telomere length in males, there was no discernible difference in the length of blood cell telomeres between female and male VPA rats. It is plausible that gender-specific regulation of the expression of certain genes encoding telomerase subunits in male rats may occur following exposure to VPA. These genes are involved in the elongation of chromosome ends through the activation of telomerase in blood cells. Previous studies have demonstrated that VPA can induce TERT activity in rat embryonic brain and cultured rat primary neural progenitor cells. However, the impact of gender on this phenomenon has not been investigated (Kim et al. 2017).

Previously, differences in telomere length between different areas of the healthy human brain were observed. However, the telomere length of different brain structures does not differ between sexes with age (Cherif et al. 2003; Mamdani et al. 2015). It is currently unclear whether these differences are preserved in the cells of the brain structures of patients with ASD. Accordingly, the present study was designed to examine the telomere length of two brain regions, namely the hippocampus and the prefrontal cortex, which have been identified as playing a pivotal role in the impairment of cognitive functions, emotional and social regulation, and spatial navigation in ASD individuals (Mohapatra & Wagner 2023; Banker et al. 2021). It was observed that telomere length in the prefrontal cortex of females in the control group and those with untreated VPA was longer than in males. Conversely, in the hippocampus, this parameter differed significantly between males and females, irrespective of the study group. A number of factors may account for this discrepancy. The shortening of telomere length is indicative of not only cellular ageing, but also neurodegeneration, as observed in autism (Kern et al. 2013). It can therefore be postulated that another reason for the observed differences in behavior may be associated with the presence of long telomeres in the prefrontal cortex of female rats and in the hippocampus of male rats, regardless of group. These differences in telomere length may be associated with differences in the function of these brain regions. The prefrontal cortex is responsible for a number of cognitive functions, including decision-making, planning, behavioral control and emotional regulation (Mohapatra & Wagner 2023). The presence of long telomeres in the prefrontal cortex of females may be indicative of a heightened level of protection for cellular DNA and an enhanced capacity for DNA repair. This may, in turn, be associated with a more efficient prefrontal cortex function in females. The hippocampus is a vital structure for memory formation and spatial navigation (Banker et al. 2021). Long telomeres in the hippocampus of male rats may indicate a better ability to preserve and repair cellular DNA, which may be associated with more efficient memory and spatial navigation in males. However, it should be noted that these links between long telomeres and brain function are speculative and require further research to be fully understood.

In order to further elucidate the discrepancy in telomere length observed in brain structures of rats subjected to an autism model, we undertook an investigation into the expression of genes that are directly responsible for telomere lengthening, namely telomerase activity (Wojtyla et al. 2011). A comparative analysis of the expression of telomerase and shelterin complex subunit genes in females and males in the valproate model of autism versus the control group revealed some significant changes, which were specific to each sex. In VPA males, the gene responsible for protecting telomeres, 1 A, *Pot1a*, exhibited increased expression exclusively in blood cells. Conversely, in females, *Pot1a* gene expression was diminished solely in the hippocampus, while another ortholog of *Pot1a* demonstrated a notable elevation in blood cells of VPA females.

As indicated on the SFARI website (https://gene.sfari.org/), this gene is a strong candidate for de novo mutations that have been observed to cause ASD in children (Prasad et al. 2012; Zhou et al. 2022; Cirnigliaro et al. 2023). In rats, this gene, which comprises two orthologs (A and B), plays a role in the shelterin complex that is distinct from its function in humans. It binds to telomeres to maintain their structural integrity and length. It has been previously demonstrated that POT1a is more efficacious in suppressing the DNA damage signal at telomeres than POT1b. Of greater significance, however, is the fact that the latter possesses the capacity to regulate the amount of single-stranded DNA at the end of telomeres (Hockemeyer et al. 2006). It is additionally noteworthy that in VPA females, the *Pot1a* expression was diminished in the hippocampus, along with another gene, Tpp1, which encodes tripeptidyl peptidase 1 and constitutes part of the shelterin complex (Sekne et al. 2022). The Pot1-Tpp1 complex has been demonstrated to bind the telomeric protrusion, thereby suppressing the ATR-dependent DNA damage response and recruiting telomerase to telomeres for DNA replication (Aramburu et al. 2020). The human POT1-TPP1 complex serves as an illustrative example of how mutations in these proteins can contribute to the development of various pathological conditions. This association has been demonstrated in several studies (Aoude et al. 2015; Bisht et al. 2016; Guo et al. 2014). The substantial alterations in *Pot1a/Pot1b-Tpp1* gene expression may, to some extent, be attributed to an endeavor to avert the impairment of cellular functions resulting from prenatal exposure to valproic acid, or alternatively, to an attempt to circumvent damage through the modulation of the telomerase enzyme. In VPA females’ model of autism, the hippocampus, which is implicated in the pathogenesis of ASD, exhibited a reduction in the expression of *Tnks*, which encodes tankyrase. The expression of the *Tnks* gene may play a role in the regulation of the cell cycle, apoptosis, cell differentiation, and other processes (Wojtyla et al. 2011; Klapper et al. 2003), which may be disrupted in autism. Therefore, increased *Tnks* gene expression in blood cells of females in the autism model may be one mechanism contributing to the changes in cellular function associated with this disorder. To fully understand the mechanisms underlying this phenomenon, additional studies are needed to elucidate the specific role of the *Tnks* gene and its relationship to autism.

Previously, only one child patient with ASD, mild facial dysmorphisms and cardiac anomalies was found to have a duplication in the TNKS gene (Corrêa et al., 2022). It is established that valproic acid, employed in the induced model of autism, can stimulate the canonical Wnt signalling pathway through modulation of histone deacetylase and GSK- 3 (Wang et al. 2015; Wiltse 2005; Go et al. 2012). This evidence demonstrates the involvement of the Wnt pathway in the pathophysiology of ASD, and potentially specifically inhibits the expression of the tankyrase gene, which is observed in females in the hippocampus. In a further existing model of ASD, the use of the tankyrase inhibitor XAV939 has been observed to result in impaired development and function of dendrites and dendritic spines of excitatory neurons, as well as alterations to the distribution of interneurons, which collectively give rise to ASD features (Fang et al. 2014).

Currently, there is a lack of comprehensive data regarding the impact of prenatal valproic acid administration on the genes associated with telomerase subunits and the shelterin complex in adult rodents. Further research is essential to explore these effects, particularly concerning the potential influences of the antidepressant amitriptyline, the nootropic agent nooclerin, and the atypical antipsychotic risperidone within this model.

The study of telomere length dynamics can provide further insight into the impact of pharmaceutical agents on cells in diverse tissues, as well as to evaluate their potential benefits and risks in the context of ASD treatment. Risperidone is an atypical antipsychotic medication that is frequently employed in the treatment of schizophrenia, bipolar disorder, and irritability associated with autism spectrum disorder (FDA Approved Drug Products: Risperdal (risperidone) for oral use).

This is the first study that risperidone increases telomere length in whole blood and prefrontal cortex cells of male VPA relative to controls. However, this increase was not observed in untreated VPA rats. The existing literature on the effect of antipsychotic drugs, including risperidone, on telomere length in peripheral blood cells is inconclusive. For example, a study by Monroy-Jaramillo et al. (2017) found that olanzapine may lead to a decrease in telomere length. Conversely, work by Yu et al. (2008) found that schizophrenia patients who responded positively to pharmacological treatment had longer telomeres. However, a study by Li et al. suggests that baseline telomere length may predict response to antipsychotic treatment (2015). Researchers from Brazil showed that the antipsychotics aripiprazole and haloperidol increased telomere length by 23% and 20% in peripheral blood mononuclear cells from healthy volunteers after acute oxidative stress injury (Polho et al. 2022). Additionally, a study examining the effects of haloperidol and clozapine on telomerase activity in peripheral blood mononuclear cells revealed no discernible impact (Porton et al. 2008). In the initial phase of the study, risperidone administration in rats resulted in notable alterations in the expression of the *Trf2* gene in the prefrontal cortex. This was observed exclusively in male subjects, where an increase in gene expression was documented, while in females, the opposite trend was evident. The *Trf2* gene, which encodes the telomere repeat binding factor 2 protein, is highly expressed in the rat brain, as evidenced by data from the Expression Atlas (https://www.ebi.ac.uk/gxa/home).

The Trf2 protein is a component of the shelterin complex, which interacts with telomeres, bending them and stabilizing the t-loop (O’Sullivan & Karlseder 2010; De Boeck et al. 2009). The Trf2 protein has been demonstrated to exert a negative regulatory effect on telomere elongation, while exhibiting no discernible impact on telomerase activity (Smogorzewska et al. 2000). In the present study, an increase in Trf1 and Trf2 gene expression in VPA males’ model of autism was associated with a relative increase in prefrontal cortex, as compared to the control group of rats (healthy). However, this increase was not observed in rats within the studied autism model. Additionally, telomere length was found to be elevated in both the VPA and control groups, as compared to the healthy control group. In females, the direction of *Trf2* gene expression differed from that observed in males, which may be attributed to a sex effect.

It has been demonstrated that risperidone, an atypical dopamine and serotonin receptor antagonist, can influence gene expression, including *Trf1* and *Trf2*, in a range of models of autism. Valproic acid is employed in the creation of an animal model of autism due to the fact that maternal administration of valproic acid during pregnancy can result in alterations to the offspring’s brain development that are analogous to certain characteristics of autism. These changes can affect genes that encode telomerase subunits, whose biological function is to maintain cell viability by regulating the cell cycle and the response to DNA damage (Smogorzewska et al. 2000; Klapper et al. 2003; Kim et al. 2017). It is plausible that risperidone may modulate *Trf2* gene expression via its impact on neurotransmitter systems and signaling pathways within the brain (Gao et al. 2024). Nevertheless, the precise mechanisms through which risperidone influences *Trf2* gene expression in the valproate rat model of autism remain to be elucidated through further investigation.

Amitriptyline is a pharmaceutical agent that has been demonstrated to be efficacious in the treatment of depressive disorders, anxiety, agitation, and sleep disturbances in children with ASD (Hellings 2023). The drug increases the levels of norepinephrine in synapses and/or serotonin in the central nervous system by inhibiting the reuptake of these mediators (Stahl 2008). As previously demonstrated, amitriptyline has been shown to increase the expression of the telomeric binding factor protein (TRF1/TRF2) in mouse spermatogenic cells (Sołek et al. 2021). In our own study, amitriptyline affected telomeres solely in relation to the increase in the indicator in VPA males’ model of autism in the prefrontal cortex. However, in VPA males, amitriptyline increased the expression of *Tep1*, *Tert*, and *Tnks* only in the blood cells. In VPA, females resulted in a notable overexpression of the *Dkc1*, *Terf1ip*, *Tnks*, *Tpp1*, and *Trf2* genes in the hippocampus and the *Pot1a* and *Tnks* genes in blood cells. In both tissues, an increase in *Tnks* expression was observed, which may demonstrate a protective role in relation to the growth and survival of different types of hippocampal neurons, affecting the Wnt signalling pathways (Wang et al. 2015; Wiltse 2005; Go et al. 2012; Fang et al. 2014).

Nooclerin (deanol aceglumate), a nootropic agent, has been demonstrated to enhance cognitive function and memory. In a randomized study, the administration of the drug to children suffering from tension headaches was observed to result in the manifestation of an anxiolytic effect (Shipilova et al., 2019). Our findings demonstrated that in males, the treatment resulted in an increase in telomere length in the blood relative to the VPA group without treatment and the control group. Additionally, it led to an increase in telomere length in the prefrontal cortex relative to the control group. In the absence of data on this drug in relation to the treatment of autism in children, and given its clinical use as an anti-anxiety drug, it can be postulated that this drug exerts a beneficial effect on telomere length in VPA males model of autism in blood cells and the prefrontal cortex. With respect to gene expression, nooclerin elicited disparate effects contingent on the sex of the animals. Therefore, in VPA males’ model of autism, nooclerin administration in the prefrontal cortex resulted in a reduction in *Trf1* expression, whereas in blood cells, it caused a threefold increase. Additionally, in the hippocampus, it led to a decrease in the *Pot1b* gene. In VPA females’ model of autism, nooclerin exerted the most pronounced influence on alterations in the transcriptional activities of the *tnks* genes, telomerase (*Tep1*, *Dkc1*) and the shelterin complex (*Terf2ip*, *Pot1a*, *Tinf2*, *Tpp1*, *Trf1 - 2*) in the hippocampus. In the prefrontal cortex of VPA females, nooclerin resulted in an increase in the expression of the *Trf2* gene, and in blood cells, a change in the expression of *Pot1a-b*, *Tinf2*, and *Tnks*.

Nooclerin is a nootropic agent that is similar in chemical structure to natural brain metabolites (GABA, glutamic acid) (Okovity et al. 2016). Glutamic acid is an amino acid and the main excitatory neurotransmitter in the central nervous system. Research shows that people with autism may have changes in glutamate metabolism, which can affect their behavior and cognitive functions (Coghlan et al. 2012). In the context of the valproate model of autism in rats, the effect of nooclerin on the expression of telomerase subunit genes and the shelterin complex can be explained by several mechanisms. Firstly, nooclerin has the capacity to interact with specific transcription factors and RNAs, thereby modulating their activity. Such alterations may result in modifications to the expression of genes linked to telomerase (e.g., *Dkc1*, *Tep1*) and the shelterin complex (e.g., *Trf1*, *Trf2*, *Tinf2*, etc.). An increase or decrease in the expression of these genes may affect telomere stability and chromosome protection (Sekne et al. 2022; Smogorzewska et al. 2000). Secondly, valproic acid, which is used in the autism model, has been demonstrated to cause oxidative stress and other forms of cellular damage (Hansen et al. 2021). It is hypothesized that nooclerin may play a role in cellular responses to stress, which may affect the expression of genes associated with DNA repair and telomere maintenance. Thirdly, nooclerin is also implicated in neuroplastic processes (Okovity S. V. et al., 2016). Such alterations in molecular processes may influence neuronal differentiation and survival, which in turn may affect the expression of genes associated with telomeres and the shelterin complex. This may be of particular importance in the context of neurodegenerative processes observed in autism (Kern et al. 2013). It can be reasonably deduced that nooclerin exerts a multitude of effects on the expression of genes encoding telomerase subunits and shelterin complex components in the valproate rat model of autism. These effects are likely to be mediated by mechanisms associated with transcriptional regulation, cellular stress response, neuroplasticity, and potentially epigenetic modifications.

The most significant alterations in gene expression were observed in females within the valproate model of autism in the hippocampus. The disparate effects of the examined pharmaceuticals on the expression of genes encoding telomeres and the shelterin complex, contingent on sex, may be attributable to the influence of estrogens, which are predominantly present in females, and which have the capacity to modulate the expression of genes associated with hippocampal function or telomere length (Kimura et al. 2004; Finney et al. 2020). Modifications in the transcriptional activity of telomerase complex genes may impact the aging processes of neurons and, subsequently, hippocampal functionality. This phenomenon may be more pronounced in females (Finney et al. 2020). Changes in the expression activity of shelterin complex genes may be critical in the context of stress and neuroplasticity, which may also explain the observed changes in the hippocampus. It is possible that in the SMA model, females are exposed to specific stress or metabolic conditions that activate these molecules, while males may respond differently, perhaps due to differences in hormonal background or other biological factors (Kimura et al. 2004; Hodes & Epperson, 2019).

In light of the contradictory findings in the existing literature and the limited quantity of available data, it remains unclear precisely how the studied drugs operate at the cellular level. Further studies are required to gain a deeper understanding of the effects of risperidone, amitriptyline and nooclerin on telomeres and the transcriptional activity of telomerase and shelterin complex genes in blood cells and brain structure.

It is important to note that this is only one aspect of the study, and a comprehensive understanding of the mechanism of action of the drugs and their impact on autism requires further research. In this study, only telomere length was measured; however, information on telomerase activity in the studied cells would provide further insight into the underlying mechanisms responsible for the observed changes in telomere length in rats. A second limitation is the insufficient number of animals in the studied groups, which could enhance the level of reliability. Thirdly, the assessment of epigenetic changes in the valproate model of autism in rats was not conducted, nor was it carried out when using drugs (risperidone, amitriptyline and nooclerin) that have the potential to affect the gene expression that was studied. Fourthly, the impact of the investigated pharmaceutical agents on telomere length and the expression of telomerase/shelterin-related genes was not evaluated in the control group. A further limitation of this study is that it did not evaluate the effect of these drugs on the lifespan of the subjects. Our current study efforts to examine the effect of risperidone and amitriptyline on the lifespan of rats exhibiting autistic-like behaviors.

## Supplementary Information

Below is the link to the electronic supplementary material.

Supplementary file1 (XLSX 33 KB)

## Data Availability

No datasets were generated or analysed during the current study.

## References

[CR1] Angoa-Pérez M, Kane MJ, Briggs DI, Francescutti DM, Kuhn DM (2013) Marble burying and nestlet shredding as tests of repetitive, compulsive-like behaviors in mice. J vis Exp 24(82):50978. 10.3791/50978 10.3791/50978 PMC4108161 24429507

[CR2] Aoude LG, Pritchard AL, Robles-Espinoza CD, Wadt K, Harland M, Choi J, Gartside M, Quesada V, Johansson P, Palmer JM, Ramsay AJ, Zhang X, Jones K, Symmons J, Holland EA, Schmid H, Bonazzi V, Woods S, Dutton-Regester K, Stark MS, Snowden H, van Doorn R, Montgomery GW, Martin NG, Keane TM, López-Otín C, Gerdes AM, Olsson H, Ingvar C, Borg A, Gruis NA, Trent JM, Jönsson G, Bishop DT, Mann GJ, Newton-Bishop JA, Brown KM, Adams DJ, Hayward NK (2015) Nonsense mutations in the shelterin complex genes ACD and TERF2IP in familial melanoma. Journal of the National Cancer Institute 107(2):dju408. 10.1093/jnci/dju408 25505254 10.1093/jnci/dju408 PMC4334787

[CR3] Aramburu T, Plucinsky S, Skordalakes E (2020) POT1-TPP1 telomere length regulation and disease. Comput Struct Biotechnol J 18:1939–1946. 10.1016/j.csbj.2020.06.040 32774788 10.1016/j.csbj.2020.06.040 PMC7385035

[CR4] Aviv A (2002) Telomeres, sex, reactive oxygen species, and human cardiovascular aging. J Mol Med 80:689–695. 10.1007/s00109-002-0374-5 12436345 10.1007/s00109-002-0377-8

[CR5] Banker SM, Pagliaccio D, Ramphal B, Thomas L, Dranovsky A, Margolis AE (2021) Altered structure and functional connectivity of the hippocampus are associated with social and mathematical difficulties in nonverbal learning disability. Hippocampus 31(1):79–88. 10.1002/hipo.23264 32949475 10.1002/hipo.23264 PMC7749072

[CR6] Barrett ELB, Richardson DS (2011) Sex differences in telomeres and lifespan. Aging Cell 10(6):913–921. 10.1111/j.1474-9726.2011.00741.x 21902801 10.1111/j.1474-9726.2011.00741.x

[CR7] Bisht K, Smith EM, Tesmer VM, Nandakumar J (2016) Structural and functional consequences of a disease mutation in the telomere protein TPP1. Proc Natl Acad Sci 113(46):13021–13026. 10.1073/pnas.1607426113 27807141 10.1073/pnas.1605685113 PMC5135350

[CR8] Blackburn EH (2005) Telomerase and cancer. Mol Cancer Res 3(4):477–482. 10.1158/1541-7786.mcr-05-0147 16179494 10.1158/1541-7786.MCR-05-0147

[CR9] Bondarenko NA (2017) Anxiety and the problem of “inattentive” animals in water maze tests. Cognitive Science in Moscow 4(4):45–51

[CR10] Buttet, M., Yu, K., Smith, J., & Johnson, R. (2022). Effect of a lifestyle intervention on telomere length: a systematic review and meta-analysis. *Mechanisms of Ageing and Development*, 111694. 10.1016/j.mad.2022.111694 10.1016/j.mad.2022.111694 35760212

[CR11] Cawthon RM (2002) Telomere measurement by quantitative PCR. Nucleic Acids Res 30(10):e47–e47. 10.1093/nar/30.10.e47 12000852 10.1093/nar/30.10.e47 PMC115301

[CR12] Cherif H, Tarry JL, Ozanne SE, Hales CN (2003) Ageing and telomeres: a study into organ-and gender-specific telomere shortening. Nucleic Acids Res 31(5):1576–1583. 10.1093/nar/gkg224 12595567 10.1093/nar/gkg208 PMC149817

[CR13] Cirnigliaro M, Chang TS, Arteaga SA, Pérez-Cano L, Ruzzo EK, Gordon A, Bicks LK, Jung JY, Lowe JK, Wall DP, Geschwind DH (2023) The contributions of rare inherited and polygenic risk to ASD in multiplex families. Proc Natl Acad Sci USA 120(31):e2215632120. 10.1073/pnas.2215632120 37506195 10.1073/pnas.2215632120 PMC10400943

[CR14] Coghlan S, Horder J, Inkster B, Mendez MA, Murphy DG, Nutt DJ (2012) GABA system dysfunction in autism and related disorders: from synapse to symptoms. Neurosci Biobehav Rev 36(9):2044–2055. 10.1016/j.neubiorev.2012.07.005 22841562 10.1016/j.neubiorev.2012.07.005 PMC4477717

[CR15] Corrêa, M. M., Corrêa, T., Santos-Rebouças, C. B., Rodrigues, M. M., de Luca, G. R., & de Camargo Pinto, L. L. (2022). New insights into candidate genes for autism spectrum disorder in 8p23.1 duplication syndrome. *Brazilian Journal of Case Reports*, 3(1), 16–23. 10.52600/2763-583X.bjcr.2023.3.1.16-23

[CR16] De Boeck G, Forsyth RG, Praet M, Hogendoorn PC (2009) Telomere-associated proteins: cross-talk between telomere maintenance and telomere-lengthening mechanisms. J Pathol 217(3):327–344. 10.1002/path.2500 19142887 10.1002/path.2500

[CR17] Dixit PV, Sahu R, Mishra DK (2020) Marble-burying behavior test as a murine model of compulsive-like behavior. J Pharmacol Toxicol Methods 102:106676. 10.1016/j.vascn.2020.106676 31954839 10.1016/j.vascn.2020.106676

[CR18] Fang WQ, Chen WW, Jiang L, Liu K, Yung WH, Fu AK, Ip NY (2014) Overproduction of upper-layer neurons in the neocortex leads to autism-like features in mice. Cell Rep 9(5):1635–1643. 10.1016/j.celrep.2014.10.022 25466248 10.1016/j.celrep.2014.11.003

[CR19] Finney CA, Rooney TP, Quattrochi CL, Wellman CL (2020) The role of hippocampal estradiol in synaptic plasticity and memory: a systematic review. Front Neuroendocrinol 56:100818. 10.1016/j.yfrne.2019.100818 31843506 10.1016/j.yfrne.2019.100818

[CR20] Frazier TW, Georgiades S, Bishop SL, Hardan AY (2014) Behavioral and cognitive characteristics of females and males with autism in the Simons Simplex Collection. J Am Acad Child Adolesc Psychiatry 53(3):329–340. 10.1016/j.jaac.2013.11.014 24565360 10.1016/j.jaac.2013.12.004 PMC3935179

[CR21] Gao S, Huang S, Xu Y, Wang B, Cheng P, Lu Y, Gilson E, Ye J (2024) Role of the telomeric factor TRF2 in post-hypoxic brain damages. Redox Biol 75:103278. 10.1016/j.redox.2024.103278 39128227 10.1016/j.redox.2024.103278 PMC11369364

[CR22] Gardner, M., Bann, D., Wiley, L., Cooper, R., Hardy, R., Nitsch, D., Martin-Ruiz, C., Shiels, P., Sayer, A.A., Barbieri, M., Bekaert, S., Bischoff, C., Brooks-Wilson, A., Chen, W., Cooper, C., Christensen, K., Meyer, T.D., Deary, I., Der, G., Roux, A. D., Fitzpatrick, A., Hajat, A., Halaschek-Wiener, J., Harris, S., Hunt, C., Jagger, C., Jeon, H.-S., Kaplan, R., Kimura, M., Lansdorp, P., Li, C., Maeda, T., Mangino, M., Nawrot, T.S., Nilsson, P., Nordfjall, K., Paolisso, G., Ren, F., Riabowol, K., Robertson, T., Roos, G., Staessen, J. A., Spector, T., Tang, N., Unryn, B., Harst, P., Woo, J., Xing, C., Yadegarfar, M. E., Park J. Y., Young, N., Kuh, D., von Zglinicki T., Ben-Shlomo Y., Halcyon Study Team (2014) Gender and telomere length: systematic review and meta-analysis. Exp Gerontol 51:15–27. 10.1016/j.exger.2013.12.004 24365661 10.1016/j.exger.2013.12.004 PMC4523138

[CR23] Go HS, Kim KC, Choi CS, Jeon SJ, Kwon KJ, Han SH, Lee J, Cheong JH, Ryu JH, Kim CH, Ko KH, Shin CY (2012) Prenatal exposure to valproic acid increases the neural progenitor cell pool and induces macrocephaly in rat brain via a mechanism involving the GSK-3β/β-catenin pathway. Neuropharmacology 63(6):1028–1041. 10.1016/j.neuropharm.2012.07.028 22841957 10.1016/j.neuropharm.2012.07.028

[CR24] Guo Y, Kartawinata M, Li J, Pickett HA, Teo J, Kilo T, Barbaro PM, Keating B, Chen Y, Tian L, Al-Odaib A, Reddel RR, Christodoulou J, Xu X, Hakonarson H, Bryan TM (2014) Inherited bone marrow failure associated with germline mutation of ACD, the gene encoding telomere protein TPP1. Blood 124(18):2767–2774. 10.1182/blood-2014-04-568508 25205116 10.1182/blood-2014-08-596445 PMC4215308

[CR25] Hansen JM, Buckland ME, Crowe A (2021) Valproic acid promotes SOD2 acetylation: a potential mechanism of valproic acid-induced oxidative stress in developing systems. Free Radical Res 55(11–12):1130–1144. 10.1080/10715762.2021.2017913 34895005 10.1080/10715762.2021.2017913

[CR26] Hellings J (2023) Pharmacotherapy in autism spectrum disorders, including promising older drugs warranting trials. World Journal of Psychiatry 13(6):262–277. 10.5498/wjp.v13.i6.262 37383284 10.5498/wjp.v13.i6.262 PMC10294139

[CR27] Hirota T, King BH (2023) Autism spectrum disorder: a review. JAMA 329(2):157–168. 10.1001/jama.2022.23661 36625807 10.1001/jama.2022.23661

[CR28] Hockemeyer D, Daniels JP, Takai H, de Lange T (2006) Recent expansion of the telomeric complex in rodents: two distinct POT1 proteins protect mouse telomeres. Cell 126(1):63–77. 10.1016/j.cell.2006.04.044 16839877 10.1016/j.cell.2006.04.044

[CR29] Hodes GE, Epperson CN (2019) Sex Differences in Vulnerability and Resilience to Stress Across the Life Span. Biological Psychiatry 86(6):421–432. 10.1016/j.biopsych.2019.04.028 31221426 10.1016/j.biopsych.2019.04.028 PMC8630768

[CR30] Ingram JL, Zorumski CF, Paul IA (2000) Prenatal exposure of rats to valproic acid reproduces the cerebellar anomalies associated with autism. Neurotoxicol Teratol 22(3):319–324. 10.1016/S0892-0362(00)00028-3 10840175 10.1016/s0892-0362(99)00083-5

[CR31] Kennedy DP, Adolphs R (2012) The social brain in psychiatric and neurological disorders. Trends Cogn Sci 16(11):559–572. 10.1016/j.tics.2012.09.006 23047070 10.1016/j.tics.2012.09.006 PMC3606817

[CR32] Kern JK, Geier DA, Sykes LK, Geier MR (2013) Evidence of neurodegeneration in autism spectrum disorder. Translational Neurodegeneration 2(1):1–6. 10.1186/2047-9158-2-17 23925007 10.1186/2047-9158-2-17 PMC3751488

[CR33] Kim KC, Choi CS, Gonzales ELT, Mabunga DFN, Lee SH, Jeon SJ, Hwangbo R, Hong M, Ryu JH, Han SH, Bahn GH, Shin CY (2017) Valproic acid induces telomerase reverse transcriptase expression during cortical development. Experimental Neurobiology 26(5):252–265. 10.5607/en.2017.26.5.252 29093634 10.5607/en.2017.26.5.252 PMC5661058

[CR34] Kim KC, Choi CS, Kim JW, Han SH, Cheong JH, Ryu JH, Shin CY (2016) MeCP2 modulates sex differences in the postsynaptic development of the valproate animal model of autism. Mol Neurobiol 53:40–56. 10.1007/s12035-015-9296-5 25404090 10.1007/s12035-014-8987-z

[CR35] Kim KC, Lee DK, Go HS, Kim P, Choi CS, Kim JW, Jeon SJ, Song MR, Shin CY (2014) Pax6-dependent cortical glutamatergic neuronal differentiation regulates autism-like behavior in prenatally valproic acid-exposed rat offspring. Mol Neurobiol 49(1):512–528. 10.1007/s12035-013-8535-2 24030726 10.1007/s12035-013-8535-2

[CR36] Kim KC, Park KH, Choi MS, Kim YH (2013) Male-specific alteration in excitatory post-synaptic development and social interaction in pre-natal valproic acid exposure model of autism spectrum disorder. J Neurochem 124(6):832–843. 10.1111/jnc.12255 23311691 10.1111/jnc.12147

[CR37] Kimura, A., Ohmichi, M., Kawagoe, J., Kyo, S., & Matsuzawa, Y. (2004). Induction of hTERT expression and phosphorylation by estrogen via Akt cascade in human ovarian cancer cell lines. *Oncogene*, 23(4505–4515). 10.1038/sj.onc.1207582 10.1038/sj.onc.1207582 15048073

[CR38] Klapper W, Qian W, Schulte C, Parwaresch R (2003) DNA damage transiently increases TRF2 mRNA expression and telomerase activity. Leukemia 17(10):2007–2015. 10.1038/sj.leu.2403086 14513051 10.1038/sj.leu.2403086

[CR39] Lansdorp PM (2022) Telomeres, telomerase and cancer. Arch Med Res 53(8):741–746. 10.1016/j.arcmed.2022.09.006 36334946 10.1016/j.arcmed.2022.10.004

[CR40] Lewis CR, Taguinod F, Jepsen WM, Cohen J, Agrawal K, Huentelman MJ, Smith CJ, Ringenbach SDR, Braden BB (2020) Telomere length and autism spectrum disorder within the family: relationships with cognition and sensory symptoms. Autism Res 13(7):1094–1101. 10.1002/aur.2307 32323911 10.1002/aur.2307

[CR41] Li Z, Tang J, Li H, Chen S, He Y, Liao Y, Wei Z, Wan G, Xiang X, Xia K, Chen X (2014) Shorter telomere length in peripheral blood leukocytes is associated with childhood autism. Sci Rep 4:7073. 10.1038/srep07073 25399515 10.1038/srep07073 PMC4233346

[CR42] Lin X, He H, Wang F, Zhang H, Wang Y (2022) Markers related to oxidative stress in peripheral blood in children with autism spectrum disorder. Research in Autism Spectrum Disorders 99:102067. 10.1016/j.rasd.2022.102067

[CR43] Liu H, Tan M, Cheng B, Wang S, Xiao L, Zhu J, Li T, Liu Y, Wang J, Zhou Y (2021) Valproic acid induces autism-like synaptic and behavioral deficits by disrupting histone acetylation of prefrontal cortex ALDH1A1 in rats. Front Neurosci 15:641284. 10.3389/fnins.2021.641284 33994921 10.3389/fnins.2021.641284 PMC8113628

[CR44] Livak KJ, Schmittgen TD (2001) Analysis of relative gene expression data using real-time quantitative PCR and the 2−ΔΔCT method. Methods 25(4):402–408. 10.1006/meth.2001.1127 11846609 10.1006/meth.2001.1262

[CR45] Maenner, M. J. (2021). Prevalence and characteristics of autism spectrum disorder among children aged 8 years—autism and developmental disabilities monitoring network, 11 sites, United States, 2018. MMWR. *Surveillance Summaries*, 70. 10.15585/mmwr.ss7001a1 10.15585/mmwr.ss7011a1 PMC8639024 34855725

[CR46] Mamdani F, Rollins B, Morgan L, Myers RM, Barchas JD, Schatzberg AF, Sequeira PA (2015) Variable telomere length across post-mortem human brain regions and specific reduction in the hippocampus of major depressive disorder. Translational psychiatry 5(9):e636–e636. 10.1038/tp.2015.125 26371764 10.1038/tp.2015.134 PMC5068804

[CR47] Mathon NF, Lloyd AC (2001) Cell senescence and cancer. Nat Rev Cancer 1:203–213. 10.1038/35011073 11902575 10.1038/35106045

[CR48] Mirabello L, Yu K, Hunt SC, Chen L, Skol AD (2010) The association of telomere length and genetic variation in telomere biology genes. Hum Mutat 31(9):1050–1058. 10.1002/humu.21220 20597107 10.1002/humu.21314 PMC2932868

[CR49] Mohapatra, A. N., & Wagner, S. (2023). The role of the prefrontal cortex in social interactions of animal models and the implications for autism spectrum disorder. *Frontiers in Psychiatry*, 14, 1205199.10.3389/fpsyt.2023.1205199 10.3389/fpsyt.2023.1205199 PMC10318347 37409155

[CR50] Monroy-Jaramillo N, Rodríguez-Agudelo Y, Aviña-Cervantes LC, Roberts DL, Velligan DI, Walss-Bass C (2017) Leukocyte telomere length in Hispanic schizophrenia patients under treatment with olanzapine. J Psychiatr Res 90:26–30. 10.1016/j.jpsychires.2017.01.002 28226264 10.1016/j.jpsychires.2017.02.007

[CR51] Mukherjee AK, Sharma S, Sengupta S, Saha D, Kumar P, Hussain T, Srivastava A, Gupta S, Roy M, Roy AG, Chowdhury S (2018) Telomere length-dependent transcription and epigenetic modifications in promoters remote from telomere ends. PLoS Genet 14(11):e1007782. 10.1371/journal.pgen.1007782 30439955 10.1371/journal.pgen.1007782 PMC6264879

[CR52] O’Sullivan RJ, Karlseder J (2010) Telomeres: protecting chromosomes against genome instability. Nat Rev Mol Cell Biol 11(3):171–181. 10.1038/nrm2845 20125188 10.1038/nrm2848 PMC2842081

[CR53] Ohashi, M., Korsakova, E., Allen, D., Lee, P., Fu, K., Vargas, B. S., Cinkornpumin, J., Salas, C., Park, J. C., Germanguz, I., Langerman, J., Chronis, C., Kuoy, E., Tran, S., Xiao, X., Pellegrini, M., Plath, K., & Lowry, W. E. (2017). Loss of MECP2 leads to telomere dysfunction and neuronal stress. *bioRxiv*, 130401. 10.1016/j.stemcr.2018.04.001 10.1016/j.stemcr.2018.04.001 PMC5995366 29742391

[CR54] Okovity, S. V., Shustov, E. B., Bolotova, V. T., & Titovich, I. A. (2016). Neuroprotective agent based bis {2-[(2E)-4-hydroxy-4-oxobut-2-enoyloxy]-N, N-diethylethanamine} butanedioate. Grew up. *Federation: Patent*, (2015118789/152588365). https://yandex.ru/patents/doc/RU2588365C1_20160627

[CR55] Panahi Y, Salasar Moghaddam F, Babaei K, Eftekhar M, Shervin Badv R, Eskandari MR, Vafaee-Shahi M, Pezeshk H, Pedram M (2023) Sexual dimorphism in telomere length in childhood autism. J Autism Dev Disord 53(5):2050–2061. 10.1007/s10803-022-05486-2 35220523 10.1007/s10803-022-05486-2

[CR56] Polho GB, Cardillo GM, Kerr DS, Chile T, Gattaz WF, Forlenza OV, Brentani HP, De-Paula VJ (2022) Antipsychotics preserve telomere length in peripheral blood mononuclear cells after acute oxidative stress injury. Neural Regen Res 17(5):1156–1160. 10.4103/1673-5374.324852 34558545 10.4103/1673-5374.324852 PMC8552857

[CR57] Polho GB, De-Paula VJ, Cardillo G, dos Santos B, Kerr DS (2015) Leukocyte telomere length in patients with schizophrenia: a meta-analysis. Schizophr Res 165(2–3):195–200. 10.1016/j.schres.2015.04.025 25975826 10.1016/j.schres.2015.04.025

[CR58] Porton B, Delisi LE, Bertisch HC, Ji F, Gordon D, Li P, Benedict MM, Greenberg WM, Kao HT (2008) Telomerase levels in schizophrenia: a preliminary study. Schizophr Res 106(2–3):242–247. 10.1016/j.schres.2008.04.003 18829263 10.1016/j.schres.2008.08.028 PMC2613190

[CR59] Prasad A, Merico D, Thiruvahindrapuram B, Wei J, Lionel AC, Sato D, Rickaby J, Lu C, Szatmari P, Roberts W, Fernandez BA, Marshall CR, Hatchwell E, Eis PS, Scherer SW (2012) A discovery resource of rare copy number variations in individuals with autism spectrum disorder. G3: Genes, Genomes, Genetics 2(12):1665–1685. 10.1534/g3.112.004689 23275889 10.1534/g3.112.004689 PMC3516488

[CR60] Ridout KK, Ridout SJ, Price LH, Sen S, Tyrka AR (2016) Depression and telomere length: a meta-analysis. J Affect Disord 191:237–247. 10.1016/j.jad.2015.11.052 26688493 10.1016/j.jad.2015.11.052 PMC4760624

[CR61] Rynkiewicz A, Schuller B, Marchi E, Piana S, Camurri A, Lassalle A, Baron-Cohen S (2016) An investigation of the “female camouflage effect” in autism using a computerized ADOS-2 and a test of sex/gender differences. Molecular Autism 7:1–8. 10.1186/s13229-016-0073-0 26798446 10.1186/s13229-016-0073-0 PMC4721191

[CR62] Schneider T, Przewłocki R (2005) Behavioral alterations in rats prenatally exposed to valproic acid: animal model of autism. Neuropsychopharmacology 30(1):80–89. 10.1038/sj.npp.1300604 15238991 10.1038/sj.npp.1300518

[CR63] Sekne Z, Ghanim GE, van Roon AM, Nguyen THD (2022) Structural basis of human telomerase recruitment by TPP1-POT1. Science 375(6585):1173–1176. 10.1126/science.abn6840 35201900 10.1126/science.abn6840 PMC7612489

[CR64] Semina II, Mukharyamova LM, Sabirov IS, Valeeva EV, Safiullina LR, Nikitin DO (2019) The current state of the problem of autism spectrum disorders—some biomedical and socio-humanitarian aspects. Kazan medical journal 100(6):918–929. 10.17750/KMJ2019-100-6-918

[CR65] Semina II, Valeeva EV, Nikitin DO, Baichurina AZ, Nikitina AV, Shilovskaya EV, Kravtsova OA (2023) Sex differences in rats with the valproate model of autism: Disturbances in social behavior and changes in Drd1 gene expression in various brain structures. Neurosci Behav Physiol 53(4):597–608. 10.1007/s11055-023-01458-w

[CR66] Sharma SR, Gonda X, Tarazi FI (2018) Autism spectrum disorder: classification, diagnosis and therapy. Pharmacol Ther 190:91–104. 10.1016/j.pharmthera.2018.05.003 29763648 10.1016/j.pharmthera.2018.05.007

[CR67] Shay JW, Wright WE (2011) Role of telomeres and telomerase in cancer. Semin Cancer Biol 21:349–353. 10.1016/j.semcancer.2011.07.001 22015685 10.1016/j.semcancer.2011.10.001 PMC3370415

[CR68] Shipilova EM, Nesterovsky YE (2019) The influence of nooclerin on the structure of sleep disturbances in children with tension headaches. Zhurnal Nevrologii i Psikhiatrii Imeni S.S Korsakova 119(11):41–46. 10.17116/jnevro201911911141 31851171 10.17116/jnevro201911911141

[CR69] Smogorzewska A, van Steensel B, Bianchi A, Oelmann S, Schaefer MR, Schnapp G, de Lange T (2000) Control of human telomere length by TRF1 and TRF2. Mol Cell Biol 20(5):1659–1668. 10.1128/MCB.20.5.1659-1668.2000 10669743 10.1128/mcb.20.5.1659-1668.2000 PMC85349

[CR70] Sołek P, Mytych J, Tabęcka-Łonczyńska A, Koziorowski M (2021) Molecular consequences of depression treatment: a potential in vitro mechanism for antidepressants-induced reprotoxic side effects. Int J Mol Sci 22(21):11855. 10.3390/ijms222111855 34769286 10.3390/ijms222111855 PMC8584852

[CR71] Solomon M, Miller M, Taylor SL, Hinshaw SP, Carter CS (2012) Autism symptoms and internalizing psychopathology in girls and boys with autism spectrum disorders. J Autism Dev Disord 42:48–59. 10.1007/s10803-011-1215-z 21442362 10.1007/s10803-011-1215-z PMC3244604

[CR72] Stahl, S. M. (2008). Classical antidepressants, serotonin selective reuptake inhibitors and noradrenergic reuptake inhibitors. In Stahl’s essential psychopharmacology: neuroscientific basis and practical application. *Cambridge: Cambridge University Press*.

[CR73] U.S. Food and Drug Administration. (2020). Risperdal (risperidone) for oral use. Retrieved from https://www.accessdata.fda.gov/drugsatfda_docs/label/2020/020272s085,020588s072,021444s058lbl.pdf

[CR74] Walf AA, Frye CA (2007) The use of the elevated plus maze as an assay of anxiety-related behavior in rodents. Nat Protoc 2(2):322–328. 10.1038/nprot.2007.44 17406592 10.1038/nprot.2007.44 PMC3623971

[CR75] Wang L, Liu Y, Li S, Long ZY, Wu YM (2015) Wnt signaling pathway participates in valproic acid-induced neuronal differentiation of neural stem cells. Int J Clin Exp Pathol 8(1):578–585 25755748 PMC4348902

[CR76] Wang Q, Zhan Y, Pedersen NL, Fang F, Hägg S (2018) Telomere length and all-cause mortality: a meta-analysis. Ageing Res Rev 48:11–20. 10.1016/j.arr.2018.09.002 30254001 10.1016/j.arr.2018.09.002

[CR77] Wiltse J (2005) Mode of action: inhibition of histone deacetylase, altering WNT-dependent gene expression, and regulation of beta-catenin—developmental effects of valproic acid. Crit Rev Toxicol 35(8–9):727–738. 10.1080/10408440590964217 16417040 10.1080/10408440591007403

[CR78] Wojtyla A, Gladych M, Rubis B (2011) Human telomerase activity regulation. Mol Biol Rep 38:3339–3349. 10.1007/s11033-010-0439-x 21086176 10.1007/s11033-010-0439-x PMC3085100

[CR79] Xu X, Hu H, Lin Y, Huang F, Ji H, Li Y, Lin S, Chen X, Duan S (2019) Differences in leukocyte telomere length between coronary heart disease and normal population: a multipopulation meta-analysis. Biomed Res Int 2019:5046867. 10.1155/2019/5046867 31198785 10.1155/2019/5046867 PMC6526555

[CR80] Yu WY, Chang HW, Lin CH, Cho CL (2008) Short telomeres in patients with chronic schizophrenia who show a poor response to treatment. J Psychiatry Neurosci 33:244–247 18592039 PMC2441885

[CR81] Zeng Z, Zhang W, Qian Y, Huang H, Wu DJH, He Z, Ye D, Mao Y, Wen C (2020) Association of telomere length with risk of rheumatoid arthritis: A meta-analysis and Mendelian randomization. Rheumatology (Oxford) 59(5):940–947. 10.1093/rheumatology/kez524 31697380 10.1093/rheumatology/kez524

[CR82] Zhang T, Sun Y, Wei J, Zhao G, Hao W, Lv Z, Chen X, Liu Y, Wei F (2023) Shorter telomere length in children with autism spectrum disorder is associated with oxidative stress. Front Psych 14:1209638. 10.3389/fpsyt.2023.1209638 10.3389/fpsyt.2023.1209638 PMC10272824 37333916

[CR83] Zhou, X., Feliciano, P., Shu, C., Wang, T., Astrovskaya, I., Hall, J. B., Obiajulu, J. U., Wright, J. R., Murali, S. C., Xu, S. X., Brueggeman, L., Thomas, T. R., Marchenko, O., Fleisch, C., Barns, S. D., Snyder, L. G., Han, B., Chang, T. S., Turner, T. N., Harvey, W. T., Nishida, A., O’Roak, B. J., Geschwind, D. H., SPARK Consortium, Michaelson, J. J., Volfovsky, N., Eichler, E. E., Shen, Y., & Chung, W. K (2022) Integrating de novo and inherited variants in 42,607 autism cases identifies mutations in new moderate-risk genes. Nat Genet 54(9):1305–1319. 10.1038/s41588-022-01148-2 35982159 10.1038/s41588-022-01148-2 PMC9470534

